# GHM method for obtaining rationalsolutions of nonlinear differential equations

**DOI:** 10.1186/s40064-015-1011-x

**Published:** 2015-06-04

**Authors:** Hector Vazquez-Leal, Arturo Sarmiento-Reyes

**Affiliations:** Facultad de Instrumentación Electrónica, Universidad Veracruzana, Cto. Gonzalo Aguirre Beltrán S/N, Xalapa, 91000 Veracruz México; National Institute for Astrophysics, Optics and Electronics, Luis Enrique Erro No. 1, Sta. Maria, Tonantzintla, 72840 Puebla México

**Keywords:** General homotopy method, Nonlinear differential equations, Epidemic model, Heat radiation, Boundary value

## Abstract

In this paper, we propose the application of the general homotopy method (GHM) to obtain rational solutions of nonlinear differential equations. It delivers a high precision representation of the nonlinear differential equation using a few linear algebraic terms. In order to assess the benefits of this proposal, three nonlinear problems are solved and compared against other semi-analytic methods or numerical methods. The obtained results show that GHM is a powerful tool, capable to generate highly accurate rational solutions.

**AMS subject classification** 34L30

## Introduction

Solving nonlinear differential equations is an important issue in sciences because many physical phenomena are modelled using such equations (Vazquez-Leal and Sarmiento-Reyes 2015). One of the most powerful methods to approximately solve nonlinear differential equations is the homotopy perturbation method (HPM) (Aminikhah 2012; Barari et al. 2008; Biazar and Eslami 2011; Biazar and Ghanbari 2012; Compean et al. 2012; El-Sayed et al. 2012; Faraz and Khan 2011; Fathizadeh et al. 2011; Filobello-Nino et al. 2012a,b; He 2004, 2009; Khan et al. 2013, 2011a,b; Mohyud-Din et al. 2012; Vazquez-Leal et al. 2012a; Wang et al. 2012). Recently, HPM method was generalized by introducing the Rational Homotopy Perturbation method (RHPM) (Vazquez-Leal 2012; Vazquez-Leal et al. 2012b), multiparameter and nonlinearities distribution HPM (Vazquez-Leal et al. 2012c), fixed-term homotopy (Vazquez-Leal et al. 2013), and the generalized homotopy method (GHM) (Vazquez-Leal 2014). Using as inspiration the RHPM method, we propose a rational expression as a particular case of application of the GHM method. In RHPM method, we consider that the approximate solution of a differential equation can be represented by the quotient of two power series of the homotopy parameter; that quotient of power series transforms the nonlinear differential equation into a series of linear differential equations. Therefore, we propose a rational GHM version of the RHPM method with the advantage of automatically obtaining the numerator and denominator of the rational solution. The main characteristic of rational version of GHM is that obtains a Taylor series of the quotient in terms of the homotopy parameter. The resulting power series is used in the same fashion like the RHPM or HPM methods, transforming a nonlinear differential equation into a series of linear differential equations. Once solved the system of differential equations, we use the results to reconstruct the original rational expression which increase the accuracy of the approximations. To assess the potential of the proposed methodology, three nonlinear problems will be solved and compared using similar methodologies or numerical methods: a nonlinear boundary valued problem (BVP) (Li and Liao 2005), a heat radiation initial valued problem (IVP) (Ganji and Rajabi 2006), and an epidemic model containing several variables (Guerrero et al. 2011).

This paper is organized as follows. In Section ‘Basic concept of GHM method’, we introduce the basic concept of the rational version of GHM method. In Section ‘Case studies’, we show the solution of three nonlinear differential equations of different kind. Numerical simulations and a discussion about the results are provided in Section ‘Numerical simulation and discussion’. Finally, a brief conclusion is given in Section ‘Conclusions’.

## Basic concept of GHM method

It can be considered that a nonlinear differential equation can be expressed as

(1) $$ L(u)+N(u)-f(r)=0, \qquad \text{where} \qquad r\in \Omega,   $$

having as boundary condition

(2) $$ B\left(u,\frac{\partial u}{\partial \eta}\right)=0, \qquad \text{where} \qquad r\in \Gamma,   $$

where *L* and *N* are a linear and a non-linear operator, respectively; *f*(*r*) is a known analytic function, *B* is a boundary operator, *Γ* is the boundary of domain *Ω*, and *∂u*/*∂η* denotes differentiation along the normal drawn outwards from *Ω* (Wang et al. 2012).

Now, a possible homotopy formulation is

(3) $$ {\begin{aligned} H(v,p)&=(1-p)\left[L(v)-L(u_{0})\right]+p(L(v)+N(v)-f(r))\\ &=0, \qquad p\in [0,1], \end{aligned}}   $$

where *u*_0_ is the initial approximation for (1) which satisfies the boundary conditions and *p* is known as the homotopy parameter. When *p*=0, (3) is reduced to a trivial equation easy to solve, and when *p*=1, (3) is reduced to the original nonlinear differential Eq. 1 (Barari et al. 2008; He 2004,2009; Khan et al. 2013; Vazquez-Leal et al. 2012a).

On one side, for the RHPM method (Vazquez-Leal 2012; Vazquez-Leal et al. 2012b), we assume that solution for (3) can be written as power series quotient of *p*

(4) $$ v=\frac{p^{0}v_{0}+p^{1}v_{1}+p^{2}v_{2}+\cdots}{1+p^{1}w_{1}+p^{2}w_{2}+\cdots},   $$

where *v*_1_,*v*_2_,… are unknown functions to be determined by the RHPM method and *w*_1_,*w*_2_,… are known (arbitrary) functions of the independent variable.

On the other side, for the GHM method (Vazquez-Leal 2014), using as reference (4), we propose the following two particular rational power series expressions

(5) $$ \begin{aligned} v=&\,\frac{p^{0}v_{0}+p^{1}v_{1}+p^{2}v_{2}+\cdots+p^{W}v_{W}}{1+p^{W+1} v_{W+1}+p^{W+2} v_{W+2}+\cdots+p^{M} v_{M}},\\ &\qquad W\geq 0 \qquad M>W, \end{aligned}   $$

where *M* represents the order of the approximation, and

(6) $$ v=\frac{p^{0}v_{0}+p^{2}v_{2}+p^{4}v_{4}+\cdots}{1+p^{1} v_{1}+p^{3} v_{3}+\cdots},   $$

where the maximum order of the power of *p* employed is considered as the order of the approximation.

In RHPM, we obtain only the unknown coefficients of the numerator because the denominator is proposed by user. However, the improvement in this work, is that GHM obtains coefficients directly for numerator and denominator. In (5), *W* represents the order of the numerator, and *M* the order of the denominator considering that the lowest power of the denominator is *W*+1. In (6), the numerator is composed by even powers and the denominator by odd powers. Here, it is important to remark that the powers of *p* of the rational expressions (5) or (6), are in fact not repeated in numerator and denominator, otherwise, the GHM will not work properly. Therefore, this is a restriction of GHM for rational expressions.

Next, we calculate Taylor series of (5) or (6), resulting

(7) $$ \begin{aligned} v=&\,p^{0}v_{0}+p^{1} g_{1}(v_{0},v_{1})+p^{2} g_{2}(v_{0},v_{1},v_{2})\\ &+p^{3} g_{3}(v_{0},v_{1},v_{2},v_{3})+\cdots, \end{aligned}   $$

where *v*_0_,*v*_1_,… are unknown functions to be determined by the GHM method and *g*_*i*_ are functions obtained by the application of Taylor series method.

Equation (7) is substituted into (3), regrouping in terms of *p*-powers and equating its coefficients to zero. The resulting system of linear differential equations is solved to obtain *v*_0_,*v*_1_,…. Next, substituting *v*_0_,*v*_1_,… into (5) or (6) and calculating the limit, when *p*→1, provides an approximate solution for (1) in the form of

(8) $$ \begin{aligned} u=&{\lim}_{p \to 1}v=\frac{v_{0}+v_{1}+v_{2}+\cdots+v_{W}}{1+ v_{W+1}+ v_{W+2}+\cdots+ v_{M}},\\ &W\geq 0 \qquad M>W, \end{aligned}   $$

or

(9) $$ u={\lim}_{p \to 1}v=\frac{v_{0}+v_{2}+ v_{4}+\cdots}{1+ v_{1}+ v_{3}+\cdots}.   $$

Usually, a low order approximation is enough to obtain a highly accurate result as depicted in the next section. A study of convergence of GHM method was reported in (Vazquez-Leal 2014).

## Case studies

In the present section, we will solve three case studies to show the utility of the GHM method to solve nonlinear differential equations.

### Nonlinear boundary value problem

As it is known, Gelfand’s equation (Li and Liao 2005) (also known as Bratu’s problem in 1D) models the chaotic dynamics in combustible gas thermal ignition. Therefore, it is important to search for accurate solutions for this equation.

The problem is expressed as

(10) $$ y^{\prime\prime}+\kappa \exp(y)=0, \qquad y(0)=0, \quad y(1)=0,   $$

where prime denotes differentiation with respect to *t* and *κ* is known as Gelfand’s parameter.

In order to ease the application of the GHM method, we approximate the exponential term by Taylor series (using five terms), resulting the approximate Gelfand’s problem

(11) $$ \begin{aligned} y^{\prime\prime}+&\,\kappa\left(1+y +\frac{1}{2} y^{2}+\frac{1}{6}y^{3}+\frac{1}{24}y^{4}\right)=0,\\ &y(0)=0, \quad y(1)=0. \end{aligned}   $$

From (11), we establish the following homotopy equations

(12) $$  \begin{aligned} (1-p)&\left(v^{\prime\prime}+nv+n\right)+p\left(v^{\prime\prime}+\kappa\left(1+v +\frac{1}{2} v^{2}+\frac{1}{6}v^{3}\right.\right.\\&\left.\left.+\frac{1}{24}v^{4}\right)\right)=0, \end{aligned}   $$

From (5), we assume that solution for (12) has the following form

(13) $$ v= \frac{v_{0}+v_{1}p}{1+v_{2}p^{2}},   $$

where Taylor series of (13) is

(14) $$ v= v_{0}+v_{1}p+v_{0}v_{2}p^{2}+\cdots.   $$

Substituting (14) into (12) and rearranging the terms of the same order of *p*, we obtain

(15) $$  \begin{aligned} \begin{array}{lll} p^{0}: & v_{0}^{\prime\prime}+\kappa=0, & v_{0}(0)=0, v_{0}(1)=0, \\\\ p^{1}: &v_{1}^{\prime\prime}+\kappa v_{0} +\kappa {v_{0}^{2}}/2+\kappa {v_{0}^{3}}/6=0, & v_{1}(0)=0, v_{1}(1)=0,\\ p^{2}: &-v_{0}v_{2}^{\prime\prime}-2v_{0}^{\prime}v_{2}^{\prime}-v_{0}^{\prime\prime}v_{2}+\kappa v_{0}v_{1}\\&+\kappa v_{1} +\kappa {v_{0}^{2}}v_{1}/2=0, & v_{2}(0)=0, v_{2}(1)=0.\\ \end{array} \end{aligned}   $$

Considering *κ*=1, we solve (15), resulting

(16) $$ \begin{array}{ll} v_{0}=& -\frac{1}{2} t(t-1), \\\\ v_{1}=& {\frac {1}{2688}} {t}^{8}-{\frac {1}{672}} {t}^{7}-{\frac {1}{480}} {t}^{6}+{\frac {11}{960}} {t}^{5}+\frac{1}{32} {t}^{4}-\frac{1}{12} {t}^{3}\\ &+{\frac {589}{13440}} t, \\\\ v_{2}=& {\frac {\delta}{3228825600 t-3228825600}},\\\\ \delta=& 31431757+4600596 {t}^{5}+32213181 {t}^{4}\\&-47167120 {t}^{2}+ 2247245 {t}^{8}-104104 {t}^{9}\\&-11791780 {t}^{3}-1471470 {t}^{7}- 9811230 {t}^{6}\\&-1650 {t}^{13}+11550 {t}^{12}+1365 {t}^{11}-158340 {t}^{10}.\\\\ \end{array}   $$

Substituting (16) into (13) and calculating the limit when *p*→1, we obtain the second order approximation

(17) $$ \begin{array}{l} u(t)= {\lim}_{p \to 1} (v) = \frac{v_{0}+v_{1}}{1+v_{2}}, \qquad t\in [0,1].\\ \end{array}   $$

### Heat radiation equation

The governing equation for heat transfer in a lumped system of combined convective-radiative heat transfers (Ganji and Rajabi 2006) is

(18) $$ \theta^{\prime}+\theta+\epsilon_{1}\theta\theta^{\prime}+\epsilon_{2}\theta^{4} =0, \qquad \theta(0)=1,   $$

where prime denotes differentiation with respect to *τ* and *ε*_1_,*ε*_2_ are parameters of the equation.

From (18) we establish the following homotopy equations

(19) $$ (1-p)\left(v^{\prime}+v\right)+p\left(v^{\prime}+v+\epsilon_{1} vv^{\prime}+\epsilon_{2} v^{4}\right)=0.   $$

From (5), we assume that solution for (19) has the following form

(20) $$ v= \frac{v_{0}+v_{1}p}{1+v_{2}p^{2}+v_{3}p^{3}},   $$

where Taylor series of (20) is

(21) $$ v= v_{0} +p v_{1} -v_{0} v_{2} p^{2}+(-v_{0} v_{3} -v_{1} v_{2}) p^{3}+\cdots \,.   $$

Substituting (21) into (19) and rearranging the terms of the same order of *p*, we obtain

(22) $$  \begin{array}{lll} p^{0}: & v_{0}^{\prime\prime}+v_{0}=0, & \quad v_{0}(0)=1, \\\\ p^{1}: &v_{1}^{\prime\prime}+v_{1}+\epsilon v_{0}v_{0}'+\epsilon_{2} {v_{0}^{4}} =0, & \quad v_{1}(0)=0,\\\\ p^{2}: &-v_{0}v_{2}^{\prime\prime}-v_{0}v_{2}-v_{0}'v_{2}+\epsilon v_{0}v_{1}' \\ &+4\epsilon_{2}{v_{0}^{3}}v_{1} =0, &\quad v_{2}(0)=0,\\\\ p^{3}: &-v_{0} v_{3}'-v_{0}' v_{3}-v_{0} v_{3} -\epsilon_{1} {v_{0}^{2}} v_{2}'\\ &+\epsilon_{1} v_{1} v_{1}'-v_{1}' v_{2}-v_{1} v_{2}-v_{1} v_{2}' \\ &-4 \epsilon_{2} {v_{0}^{4}} v_{2}-2 \epsilon_{1} v_{0} v_{0}' v_{2}+6 \epsilon_{2} {v_{0}^{2}} {v_{1}^{2}}=0, & \quad v_{3}(0)=0. \end{array}   $$

Then, we solve (22), resulting

(23) $$  \begin{array}{ll} v_{0}=& \exp(-\tau),\\ v_{1}=& \left(-\epsilon_{1} \exp(-\tau)\,+\,\frac{1}{3} \epsilon_{2} \exp(-3 \tau)+\epsilon_{1}-\frac{1}{3} \epsilon_{2}\right) \exp(-\tau),\\ v_{2}=& \frac{1}{36}\! \left(-48 \epsilon_{1} \epsilon_{2}\,+\,16 {\epsilon_{2}^{2}}\right) \exp(-3 \tau)+\frac{1}{36} \left(72 {\epsilon_{1}^{2}}-24 \epsilon_{1} \epsilon_{2}\right)\\&\exp(-\tau) -\frac{3}{2} {\epsilon_{1}^{2}} \exp(-2 \tau)+\frac{17}{12} \epsilon_{1} \epsilon_{2} \exp(-4 \tau)-\frac{1}{2} {\epsilon_{1}^{2}}\\&+\frac{7}{12} \epsilon_{1} \epsilon_{2} -\frac{2}{9} {\epsilon_{2}^{2}} \exp(-6 \tau)-\frac{2}{9} {\epsilon_{2}^{2}},\\ v_{3}=& -\frac{8}{81} {\epsilon_{2}^{3}} \exp(-9 \tau)+\frac{253}{252} \epsilon_{1} {\epsilon_{2}^{2}} \exp(-7 \tau)+\left(-\frac{8}{9} \epsilon_{1} {\epsilon_{2}^{2}}\right.\\& +\left.\frac{8}{27} {\epsilon_{2}^{3}}\right) \exp(-6 \tau)-\frac{31}{12} \epsilon_{2} \exp(-5 \tau) {\epsilon_{1}^{2}}+\left(-\frac{11}{9} \epsilon_{1} {\epsilon_{2}^{2}}\right.\\& +\left.\frac{11}{3} {\epsilon_{1}^{2}} \epsilon_{2}\right) \exp(-4 \tau)+\left(\frac{7}{6} {\epsilon_{1}^{3}}-\frac{7}{6} {\epsilon_{1}^{2}} \epsilon_{2}-\frac{8}{27} {\epsilon_{2}^{3}}\right.\\& +\left.\!\frac{37}{36} \epsilon_{1} {\epsilon_{2}^{2}}\right) \!\exp(-3 \tau)\,+\,\left(\frac{1}{3} {\epsilon_{1}^{2}} \epsilon_{2}-{\epsilon_{1}^{3}}\right) \exp(-2 \tau)+\frac{1}{3} {\epsilon_{1}^{3}}\\& +\left(\frac{1}{12} {\epsilon_{1}^{2}} \epsilon_{2}-\frac{1}{2} {\epsilon_{1}^{3}}+\frac{1}{9} \epsilon_{1} {\epsilon_{2}^{2}}\right) \exp(-\tau)\\& +\frac{8}{81} {\epsilon_{2}^{3}}-\frac{2}{63} \epsilon_{1} {\epsilon_{2}^{2}}-\frac{1}{3} {\epsilon_{1}^{2}} \epsilon_{2}. \end{array}   $$

Substituting (23) into (20), and calculating the limit when *p*→1, we obtain the third order approximation

(24) $$ \begin{array}{ll} u(\tau)=& \frac{v_{0}+v_{1}}{1+v_{2}+v_{3}}. \\ \end{array}   $$

### Model for evolution of smoking habit in Spain

Recently, a model that describes the evolution of the smoking habit in Spain has been presented (Guerrero et al. 2011; Vazquez-Leal and Guerrero 2014). The system of four equations is

(25) $$ \begin{array}{ll} \dot{n}-\mu (1-n) +\beta n (s+c)=0, \\ \dot{s}-\beta n (s+c)-\rho e -\alpha c + (\gamma+\lambda+\mu) s=0,\\ \dot{c}- \gamma s+(\alpha +\delta +\mu)c=0,\\ \dot{e}- \lambda s - \delta c +(\rho+\mu)e=0,\\ \end{array}   $$

where dots denote differentiation with respect to *t*.

The sub-populations included in the model are: *n* is the proportion of the total population who has never smoked, *s* is the proportion of people who smoke less than 20 cigarettes per day, *c* is the proportion of individuals who smoke more than 20 cigarettes per day, and *e* is the proportion of ex-smokers.

Parameter *μ* denotes birth rate in Spain; *β* denotes the transmission rate due to social pressure to adopt smoking habit; *ρ* express the rate at which ex-smokers return to smoking; *α* is the rate at which an excessive smoker becomes a normal smoker by decreasing the number of cigarettes per day; *γ* is the rate at which normal smokers become excessive smokers by increasing the number of cigarettes per day; *λ* denotes the rate at which normal smokers stop smoking, and *δ* is the rate at which excessive smokers stop smoking.

The population is constant and it has been normalized to unity, then

(26) $$ n+s+c+e=1,   $$

for any instant of time.

We set the values of the parameters as reported in (Guerrero et al. 2013) for Spain: *μ*=0.01 years ^−1^, *ρ*=0.0425 years ^−1^, *β*=0.0381 years ^−1^, *α*=0.1244 years ^−1^, *γ*=0.1175 years ^−1^, *λ*=0.0498 years ^−1^ and *δ*=0.0498 years ^−1^. Moreover, the initial conditions are chosen as: *n*(0)=0.5045, *s*(0)=0.2059, *c*(0)=0.1559, and *e*(0)=0.1337, as reported in (Guerrero et al. 2013).

According to the GHM (relation (3)), we can construct the homotopy map as follows

(27) $$  \begin{array}{ll} (1\,-\,p)(\dot{v}_{1}-\dot{n}_{0})+\hbar p\left(\dot{v}_{1}-\mu (1\,-\,v_{1})\! +\!\beta v_{1} (v_{2}+v_{3}) \right)=0, \\ (1-p)(\dot{v}_{2}-\dot{s}_{0})+\hbar p\left(\dot{v}_{2}-\beta v_{1} (v_{2}+v_{3})-\rho v_{4} -\alpha v_{3} \right.\\\quad\left.+ (\gamma+\lambda+\mu) v_{2}\right)=0, \\ (1-p)(\dot{v}_{3}-\dot{c}_{0})+\hbar p(\dot{v}_{3}- \gamma v_{2}+(\alpha +\delta +\mu)v_{3})=0, \\ (1-p)(\dot{v}_{4}-\dot{e}_{0})+\hbar p(\dot{v}_{4}- \lambda v_{2} - \delta v_{3} +(\rho+\mu)v_{4})=0, \\ \end{array}   $$

where dots denote differentiation with respect to *t* and $\hbar $ is a control parameter. Initial approximations are

(28) $$ \begin{array}{ll} v_{1,0}(t)=n_{0}(t)=n(0)=r_{1}, \\ v_{2,0}(t)=s_{0}(t)=s(0)=r_{2}, \\ v_{3,0}(t)=c_{0}(t)=c(0)=r_{3}. \\ v_{4,0}(t)=e_{0}(t)=e(0)=r_{4}. \\ \end{array}   $$

From (6), we assume that the solution for (27) can be written as

(29) $$ \begin{array}{l} v_{i}= \frac{\sum_{j=0}^{Q} p^{2j}v_{i,2j}}{1+\sum_{j=1}^{Q} p^{2j-1}v_{i,2j-1}}, \qquad i=1,2,3,4, \end{array}   $$

where the order of the approximation is choose as 2*Q*=12.

Then, Taylor series of order 2*Q*+1 is calculated, resulting

(30) $$ \begin{array}{l} v_{i}=v_{i,0}-v_{i,0}v_{i,1}p+\left(v_{i,2}+v_{i,0}v_{i,1}^{2}\right)p^{2}+\cdots\\\hspace{19pt} +\,p^{2Q}(\cdots), \hspace{25pt} i=1,2,3,4, \end{array}   $$

where *v*_*i*,*j*_ (*i*,*j*=1,2,3,…,2*Q*) are functions yet to be determined. Substituting (30) into (27) and rearranging the coefficients of *p*-power, we have

(31) $$ \begin{array}{l} \dot{v}_{1,0}+\left(-v_{1,0} \dot{v}_{1,1}+(-1+\hbar-v_{1,1}) \dot{v}_{1,0}\right.\\\hspace{16.2pt} \left.+\hbar ((\mu+v_{2,0} \beta+v_{3,0} \beta) v_{1,0}-\mu)\right)p+\cdots=0, \\ \dot{v}_{2,0}+\left(-v_{2,0} \dot{v}_{2,1}+(-v_{2,1}-1+\hbar) \dot{v}_{2,0}\right.\\ \hspace{16.2pt}+((\gamma+\lambda+\mu-\beta v_{1,0}) v_{2,0}-\beta v_{1,0} v_{3,0}-\rho v_{3,0}\\ \hspace{16.2pt}-\alpha v_{3,0}) \left.\hbar \right)p+\cdots=0, \\\\ \dot{v}_{3,0}+ \left(-v_{3,0} \dot{v}_{3,1}+(-1+\hbar-v_{3,1}) \dot{v}_{3,0}\right.\\\hspace{16.2pt}\left.+\hbar ((\alpha+\delta+\mu) v_{3,0}-\gamma v_{2,0}) \right)p+\cdots=0. \\\\ \dot{v}_{4,0}+ (-v_{4,0} \dot{v}_{4,1}+(-v_{4,1}+\hbar-1) \dot{v}_{4,0}+((\rho+\mu) v_{4,0}\\\hspace{16.2pt}-\delta v_{3,0}-\lambda v_{2,0}) \hbar)p+\cdots=0. \\\\ \end{array}   $$

In order to obtain the unknowns *v*_*i*,*j*_(*t*) (*i*,*j*=1,2,3,…), we must construct and solve the following system of equations, considering the initial conditions of *v*_*i*,*j*_(0)=0 for *i*,*j*=1,2,3,…

(32) $$ \begin{array}{l} \dot{v}_{1,0}=0, \\ -v_{1,0} \dot{v}_{1,1}+(-1+\hbar-v_{1,1}) \dot{v}_{1,0}+\hbar ((\mu+v_{2,0} \beta\\+v_{3,0} \beta) v_{1,0}-\mu)=0, \\ \qquad \vdots \\ \dot{v}_{2,0}=0,\\ -v_{2,0} \dot{v}_{2,1}+\left(-v_{2,1}-1+\hbar\right) \dot{v}_{2,0}+((\gamma+\lambda\\+\mu-\beta v_{1,0}) v_{2,0}-\beta v_{1,0} v_{3,0}-\rho v_{3,0}-\alpha v_{3,0}) \hbar=0,\\ \qquad \vdots \\ \dot{v}_{3,0}=0,\\ -v_{3,0} \dot{v}_{3,1}+(-1+\hbar-v_{3,1}) \dot{v}_{3,0}+\hbar ((\alpha+\delta+\mu) v_{3,0}\\-\gamma v_{2,0})=0,\\ \qquad \vdots \\ \dot{v}_{4,0}=0,\\ -v_{4,0} \dot{v}_{4,1}+(-v_{4,1}+\hbar-1) \dot{v}_{4,0}+((\rho+\mu) v_{4,0}-\delta v_{3,0}\\-\lambda v_{2,0}) \hbar=0,\\ \end{array}   $$

Therefore

(33) $$  \begin{array}{l} v_{1,0}(t) =n_{0}(t)= r_{1},\\ v_{1,1}(t) =(\beta (r_{3}+r_{2})+\mu(1- 1/r_{1})) t \hbar,\\ \qquad \vdots \\ v_{2,0}(t) =s_{0}(t)= r_{2}, \\ v_{2,1}(t) =((- \beta r_{1}- \alpha) r_{3}/r_{2}\,+\,\lambda\,+\,\gamma\,+\,\mu- \beta r_{1}- r_{4} \rho/r_{2}) t \hbar,\\ \qquad \vdots\\ v_{3,0}(t)=c_{0}(t)=r_{3},\\ v_{3,1}(t)=(- \gamma r_{2}/r_{3}+\mu+\delta+\alpha) t \hbar,\\ \qquad \vdots\\ v_{4,0}(t)=e_{0}(t)=r_{4},\\ v_{4,1}(t)=(\rho+\mu+(- \lambda r_{2}- \delta r_{3})/r_{4}) t \hbar,\\ \qquad \vdots \end{array}   $$

We obtained *v*_1,3_,*v*_2,3_, *v*_3,3_, *v*_4,3_, and the succeeding terms; nevertheless, because they are too cumbersome, we skip them and will be used only in the final results. Now, from (29), we obtain a 12-th order approximation; then, considering *p*→1, yields the approximate solution for (25) as

(34) $$ \begin{array}{l} V_{i}= {\lim}_{p \to 1} v_{i}= \frac{\sum_{j=0}^{6} v_{i,2j}}{1+\sum_{j=1}^{6} v_{i,2j-1}}, \qquad i=1,2,3,4, \end{array}   $$

where *n*(*t*)=*V*_1_, *s*(*t*)=*V*_2_, *c*(*t*)=*V*_3_, and *n*(*t*)=*V*_4_.

Now, we need to determine the value of the parameter $\hbar $ to obtain the best fit for the exact solution (25).

First, we obtain the Mean Square Error (*E*_*m*_), defined as

(35) $$  \begin{array}{ll} E_{m} =& \frac{1}{K} \sum\limits^{K}_{j=0} \left\{ \!\left[ n(j \Delta t)-n_{r}(j \Delta t) \right]^{2} + \left[ s(j \Delta t)-s_{r}(j \Delta t) \right]^{2}\right.\\ & \left. + \left[ c(j \Delta t)-c_{r}(j \Delta t) \right]^{2} + \left[ e(j \Delta t)-s_{r}(j \Delta t) \right]^{2} \right\}, \end{array}   $$

where *K*=500, *Δt*=0.1; *n*_*r*_(·), *s*_*r*_(·), *c*_*r*_(·), and *e*_*r*_(·) are the numerical values obtained using the Fehlberg fourth-fifth order Runge-Kutta method with degree four interpolant (RKF45) (Enright et al. 1986; Fehlberg 1970) solution (built-in function of Maple software). We considered an absolute error of 10^−12^ for the setup.

**Fig. 1:**
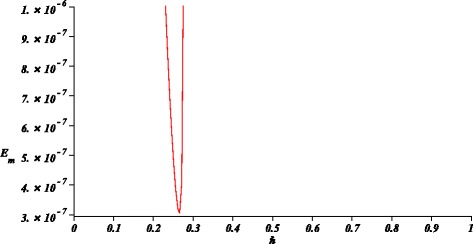
Error *E*_*m*_ versus $\hbar $ calculated using (35)

This means that *E*_*m*_ is the residual error due to the difference between the GHM solution and the exact solution within the interval 0≤*t*≤50 years. Therefore, Fig. 1 shows the minimum mean square error that corresponds, approximately, to $\hbar =0.265$. Hence, we obtain

(36) $$ \begin{array}{l} n(t)= n_{n}/n_{d}, \\ n_{n}= 0.5045- 8.2592122\times 10^{-4}t + 4.7606095\\\qquad\times 10^{-5}{t}^{2}- 1.0838343\times 10^{-6}{t}^{3} + 1.4940822\\\qquad\times 10^{-8}{t}^{4}- 1.1878388\times 10^{-10}{t}^{5}+ 4.6583518\\\qquad\times 10^{-13}{t}^{6} +{3.4582175\times 10^{-16}}{t}^{7}-1.3919721\\\qquad\times 10^{-17}{t}^{ 8}+{ 7.4384156\times 10^{-20}}{t}^{9}- 2.0139501\\\qquad\times 10^{-22}{ t}^{10}+{ 2.8243714\times 10^{-25}}{t}^{11}- 1.5671658\\\qquad\times 10^{-28 }{t}^{12},\\ n_{d}=1+ 0.0022273587t- 8.7277969\times 10^{-5}{t}^{2}\\\qquad+ 2.0224303\times 10^{-6}{t}^{3}- 2.5537127\times 10^{-8}{t}^{4}\\\qquad+ 1.8449207\times 10^{-10}{t}^{5}-{ 6.3603998 \times 10^{-13}}{t}^{6}\\\qquad -{ 3.9653010\times 10^{-16}}{t}^{7}+{ 1.3106707\times 10^{-17}}{t}^{8}\\\qquad-{5.3438537\times 10^{-20}}{t}^{ 9}+{ 9.8379312\times 10^{-23}}{t}^{10}\\\qquad-{7.1089183\times 10^{-26}} {t}^{11}, \end{array}   $$

(37) $$ \begin{array}{l} s(t)= s_{n}/s_{d}, \\ s_{n}= 0.2059- 0.0018488493t+ 8.3523428\times 10^{-5}{t}^{2}\\\qquad- 2.2529388\times 10^{-6} {t}^{3}+ 5.6365667\times 10^{-8}{t}^{4}\\\qquad- 1.1087427\times 10^{-9}{t}^{5}+{ 1.1295079\times 10^{-11}}{t}^{6}\\\qquad+{2.4934539\times 10^{-14}}{t}^{ 7}-{ 1.9822222\times 10^{-15}}{t}^{8}\\\qquad+{2.1647907\times 10^{-17}}{ t}^{9}-{ 1.0696407\times 10^{-19}}{t}^{10}\\\qquad+{ 2.2580400\times 10^{-22 }}{t}^{11}- 9.1023225\\\qquad\times 10^{-26}{t}^{12},\\ s_{d}=1+ 0.012216812t- 4.0274518\times 10^{-4}{t}^{2}\\\qquad+ 1.1094910\times 10^{-5}{t}^{3}- 2.5292158\times 10^{-7}{t}^{4}\\\qquad+ 4.5595306\times 10^{-9}{t}^{5}-{ 4.3695722 \times 10^{-11}}{t}^{6}\\\qquad -{ 2.5909092\times 10^{-14}}{t}^{7}+ 4.7726975\times 10^{-15}{t}^{8}\\\qquad-{4.3013487\times 10^{-17}}{t}^{9}+{ 1.5225447\times 10^{-19}}{t}^{10}\\\qquad-{1.7433203\times 10^{-22}} {t}^{11}, \end{array}   $$

(38) $$ {\fontsize{9.2}{6}\begin{aligned} \begin{array}{l} c(t)= c_{n}/c_{d}, \\ c_{n}= 0.1559- 0.0018686738t+ 3.0147481\times 10^{-5}{t}^{2}\\\qquad+ 1.4921561 \times 10^{-6}{t}^{3} - 7.2745605\times 10^{-8}{t}^{4}\\\qquad+ 1.4000191\times 10^{-9}{t}^{5}-{ 1.7423707\times 10^{-11}}{t}^{6} \\\qquad+{ 2.1726271\times 10^{-13}}{t}^{ 7}-{ 2.7865047\times 10^{-15}}{t}^{8}\\\qquad+{ 2.5689253\times 10^{-17}}{ t}^{9} -{ 1.4582984\times 10^{-19}}{t}^{10}\\\qquad+{ 4.8992736\times 10^{-22 }}{t}^{11}-{ 7.8167718\times 10^{-25}}{t}^{12},\\ c_{d}=1+ 0.016307979t- 2.3394796\times 10^{-4}{t}^{2}\\\qquad- 8.1415825\times 10^{-6}{t}^{3}+ 4.2775216\times 10^{-7}{t}^{4}\\\qquad- 8.1604833\times 10^{-9}{t}^{5}+{ 9.3265962 \times 10^{-11}}{t}^{6}\\\qquad-{ 9.5313836\times 10^{-13}}{t}^{7}+{ 9.8754118\times 10^{-15}}{t}^{8}\\\qquad-{ 7.3067484\times 10^{-17}}{t}^{ 9}+{ 2.9521682\times 10^{-19}}{t}^{10}\\\qquad-{ 5.2937190\times 10^{-22}} {t}^{11}, \end{array} \end{aligned}}   $$

and

$$ \begin{array}{l} e(t)= e_{n}/e_{d}, \\ e_{n}= 0.1337+ 0.0045434452t- 5.0496669\times 10^{-4}{t}^{2}\\\qquad+ 0.000029362922 {t}^{3}- 1.4018380\times 10^{-6}{t}^{4}\\\qquad+5.1516170\times 10^{-8}{t}^{5}- 1.4400901\times 10^{-9}{t}^{6}\\\qquad+{ 2.9965728\times 10^{-11}}{t}^{7}-{ 4.5474832\times 10^{-13}}{t}^{8}\\\qquad+{ 4.8775075\times 10^{-15}}{t}^{ 9}-{ 3.5078219\times 10^{-17}}{t}^{10}\\\qquad+{ 1.5229255\times 10^{-19}} {t}^{11}-{ 3.0439772\times 10^{-22}}{t}^{12}, \end{array} $$

(39) $$  \begin{array}{l} e_{d}=1- 0.046234542t+ 0.0029901252{t}^{2}- 1.6907004\\\qquad\times 10^{-4}{t}^{3}+ 7.082106\times 10^{-6}{t}^{4}- 2.2407423\\\qquad\times 10^{-7}{t}^{5}+ 5.1987627\times 10^{-9}{t}^{6}- 8.6779824\\\qquad\times 10^{-11}{t}^{7}+{ 1.0113549\times 10^{-12}}{t}^{8}- 7.8296360\\\qquad\times 10^{-15}{t}^{ 9}+{ 3.6341190\times 10^{-17}}{t}^{10}- 7.7442950\\\qquad\times 10^{-20} {t}^{11}. \end{array}   $$

## Numerical simulation and discussion

For all case studies, we used built-in numerical routines from Maple 13 for comparison purposes. For the BVP problem, it was utilized the scheme based on trapezoid combined with Richardson extrapolation. For the IVP problems, it was used the Fehlberg fourth-fifth order Runge-Kutta method with degree four interpolant (RKF45) (Enright et al. 1986; Fehlberg 1970). For both types of algorithms, it was used a tolerance of absolute error (A.E.) of 10^−12^.

**Fig. 2:**
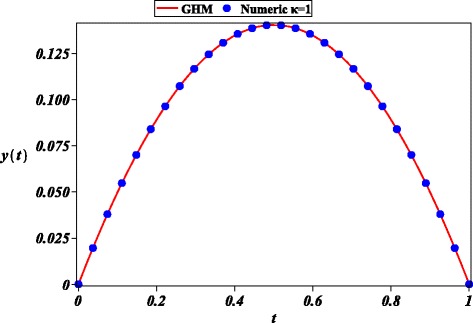
Numerical solution for (10) (solid circle) and its approximate GHM solution (17) (solid line) for different *κ*=1

**Fig. 3:**
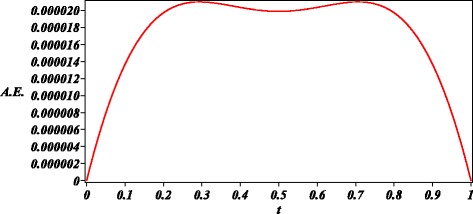
Absolute error (A.E.) of (17) with respect to numerical solution for (10)

We obtained a highly accurate approximate solution (17) for the nonlinear BVP Geldand’s problem (second order) (Li and Liao 2005) (10) as depicted in Figs. 2 and 3. Thus, the GHM method can be useful for such kind of problems that are commonly found in the area of Physics.

**Fig. 4:**
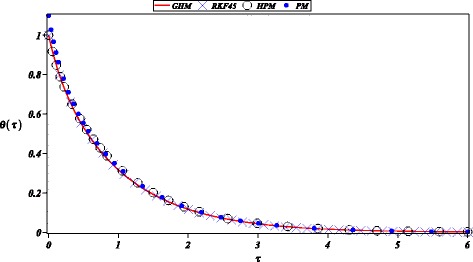
Numerical solution for (18) (diagonal cross) and its approximations obtained by: GHM (24) (solid line), HPM (Ganji and Rajabi 2006), and PM (Ganji and Rajabi 2006) (solid circle)

**Fig. 5:**
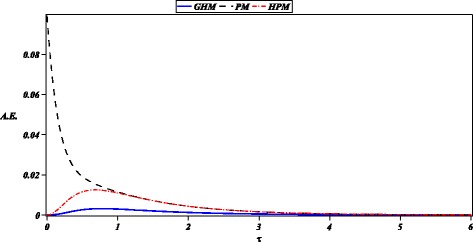
Absolute error (A.E.) of approximations GHM (24) (solid line), HPM (Ganji and Rajabi 2006) (dash-dot), and PM (Ganji and Rajabi 2006) (dash) with RKF45 solution for (18)

Additionally, we solved the heat radiation equation (18) obtaining a highly accurate solution as depicted in Figs. 4 and 5. In the same figure, we can observe a comparison between HPM (Ganji and Rajabi 2006), PM (Ganji and Rajabi 2006); noticing higher precision by the proposed solution. The high precision of GHM method is due to its ability to produce rich rational expressions that can, potentially, fit a wider scope of non-linearities. For instance, it is well know that Padé approximants (Bararnia et al. 2012; Guerrero et al. 2013; Raftari and Yildirim 2011; Torabi and Yaghoobi 2011), being rational expressions, can represent more efficiently some approximate solutions than simple series solutions.

**Fig. 6:**
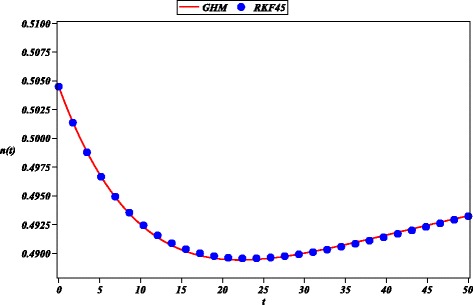
Solution of GHM for *n*(*t*) (solid line), RKF45 solution (solid circle) for (25)

**Fig. 7:**
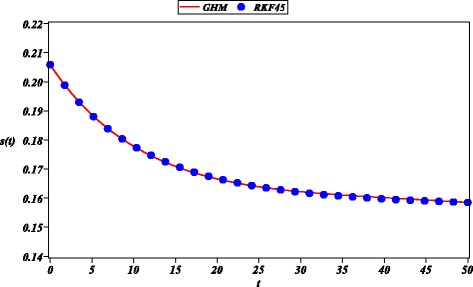
Solution of GHM for *s*(*t*) (solid line), RKF45 solution (solid circle) for (25)

**Fig. 8:**
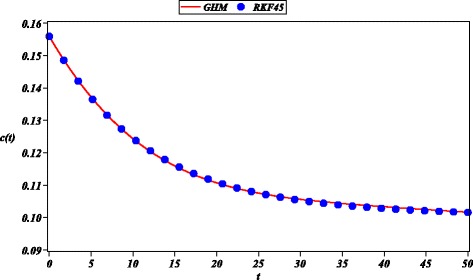
Solution of GHM for *c*(*t*) (solid line), RKF45 solution (solid circle) for (25)

**Fig. 9:**
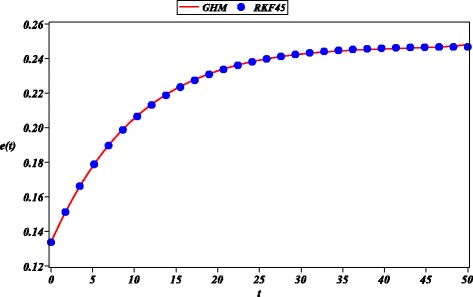
Solution of GHM for *e*(*t*) (solid line), RKF45 solution (solid circle) for (25)

Next, we approximated the multi-variable model (25) for the evolution of the smoking habit in Spain (Guerrero et al. 2011). Resulting approximations (36)-(39) are in good agreement to numerical results (RKF45) for a period of 50 years (See Figs. 6, 7, 8 and 9). Comparing Fig. 7 of this work and Fig. 6 of a HAM solution reported in (Guerrero et al. 2013), we can observe that the 12-th order GHM solution (37) possesses wider domain of convergence than the 20-th order HAM approximation (*s*(*t*)). A control of convergence $\hbar $ is employed to increase accuracy of the solution; it is done in similar fashion to the control of convergence for HAM method (Guerrero et al. 2013; Li and Liao 2005).

Thus, GHM method can potentially generate higher accurate solutions in comparison than the well established HAM method. In this example, we used the rational series (6), instead of (5), to show the flexibility of the GHM method. In fact, as long as the Taylor series of the proposed rational series keeps the general form (7), we can propose other combinations of *p*-powers to obtain more accurate solutions. In order to extend the convergence of GHM method, this method may be combined with others methods like those reported for HPM or HAM: the nonlinearities distribution homotopy perturbation method (NDHPM) (Vazquez-Leal et al. 2012c), the variational homotopy perturbation method (Matinfar et al. 2011; Noor and Mohyud-Din 2008), Padé approximants (Bararnia et al. 2012; Guerrero et al. 2013; Raftari and Yildirim 2011; Torabi and Yaghoobi 2011), Laplace-Padé after-treatment (Bahuguna et al. 2009; Ebaid 2011; Gökdoğgan et al. 2012; Jiao et al. 2002; Khan and Faraz 2011; Merdan et al. 2011; Momani and Ertürk 2008; Merdan et al. 2009; Sweilam et al. 2009; Tsai and Chen 2010), fixed-term homotopy (Vazquez-Leal et al. 2013), among others.

GHM and RHPM methods can generate highly accurate rational solutions. Nonetheless, on one side, RHPM (Vazquez-Leal 2012; Vazquez-Leal et al. 2012b) requires the proposal of an arbitrary power series for the divisor. Therefore, the RHPM solution procedure calculates only the numerator. This feature implies the requirement of adjustment parameters that should be recalculated for each specific value of the parameters of the nonlinear problem under study. On the other side, the rational version of GHM method obtains automatically the solutions for numerator and denominator. Therefore, this characteristic converts the GHM method into a more attractive tool due to its ability to generate general solutions.

The case studies where chosen in order to test the ability of GHM for the solution of problems with different type of nonlinearities and boundary conditions. For instance, the first case study exhibits an exponential nonlinearity and boundary valued conditions. Next, second case study is an initial condition problem with a four order power nonlinearity. Finally, in the last case study, we show that GHM can be applied to solve a system of nonlinear differential equations with initial conditions. In the present work, we choose arbitrary order approximations to depict the basic procedure of GHM for rational solutions, resulting highly accurate solutions (see Figs. 1, 3, and 5). In the same fashion as HPM, increasing the order of the GHM approximations will increase the accuracy. However, it is important to highlight that future work is required in order to propose a systematic procedure to choose the order of the GHM rational approximations.

In this manuscript, GHM is presented as a novel tool to find rational solutions of different nonlinear differential equations. For instance, we can observe that (24) is expressed in terms of the division of the sum of exponential terms (see (23)) and the coefficients (*ε*_1_ and *ε*_2_) of (18); this type of approximation is indeed very difficult to obtain (or impossible) with HPM, PM, HAM, among other approximative methods. Thereupon, further research is required to explore all the potential benefits of this proposal.

## Conclusions

This work introduced a rational version of the generalized homotopy method (GHM) as a useful tool with high potential to solve nonlinear differential equations. We were able to obtain accurate and handy rational solutions for different types of problems: a nonlinear BVP problem, a highly nonlinear IVP problem, and an epidemic model. The high precision of the GHM solutions is due to the generated rational expressions that can potentially fit a wider scope of non-linearities. Also, a comparison between the results of applying the proposed method and PM/HPM/HAM was shown; concluding that GHM method provided more accurate approximations. Finally, further research can be focused on the application of rational version of GHM method for the solution of nonlinear differential algebraic equations, nonlinear fractional differential equations, nonlinear partial differential equations, among others.
